# Results of surgical treatment of Hoffa fractures in pediatric population: 8-case series

**DOI:** 10.3389/fped.2025.1552420

**Published:** 2025-03-10

**Authors:** Yuan Xiao, Clement Arthur, Xin Liu

**Affiliations:** ^1^ Department of Pediatric Orthopaedics, Sichuan Provincial Orthopaedic Hospital, Chengdu, Sichuan, China; ^2^ Department of Otolaryngology-Head & Neck Surgery, First Hospital of Shanxi Medical University, Taiyuan, Shanxi, China

**Keywords:** surgical intervention, Hoffa fracture, pediatric surgery procedure, parapatellar approach, Letennuer score

## Abstract

**Objectives:**

Hoffa's fractures are extremely rare in children. Very few cases have been published in connection with this condition. The present study highlights the healing outcomes of surgical treatment in pediatric Hoffa's fractures without direct comparison to conservative treatment.

**Methods:**

During this interval, on average, eight children with Hoffa fractures were treated in our department for 10.1 years. Seven had unicondylar fractures (4 lateral and 3 medial), and one had a bicondylar fracture. Unicondylar cases were operated upon with the lateral parapatellar approach, and a combination of direct lateral and medial access with PPA was applied for the bicondylar fracture. A Cannulated Compression Screw was used for fixation. The postoperative care included restricted weight-bearing for 10 weeks and removal of the fixation at 6 months. Follow-up was conducted on knee function and pain, and Letenneur scores were evaluated.

**Results:**

The bone union was obtained between 12 and 18 weeks. In the unicondylar cases, knee function was satisfactory, and most outcome measures showed supporting results. There was limited mobility in the bicondylar case and some mild varus; the Letenneur score was fair. Unicondylar fractures with bedding and early functional exercises did well in the long term, while the results of bicondylar fractures were poor.

**Conclusion:**

This study presents a surgical treatment approach for pediatric Hoffa fractures and monitors the results. However, it does not provide a comparison with conservative measures. The findings also offer insight into the surgical protocols needed for better long-term outcomes in children with Hoffa fractures.

## Introduction

Hoffa fractures are intra-articular distal femur fractures affecting the unicondylar or bicondylar regions. These fractures are rare in adults and children (1–3). Typically resulting from high-energy trauma, Hoffa fractures can severely impact the growth and development of the distal femur in children. Studies often highlight complications associated with Hoffa fractures, including avascular necrosis of the femoral condyle (AVN), nonunion, and restricted knee mobility (4, 5). Albert Hoffa first described Hoffa fractures in 1904, characterizing them as intra-articular fractures within the coronal plane involving a single condyle (6, 7).

In understanding the pediatric implications of these fractures, this study conducted a systematic review of databases like PubMed, Scopus, and Google Scholar using keywords such as “Hoffa Fracture,” “Pediatric,” “Children,” and “Coronal Fracture of Femoral Condyle.” In over fifty relevant pieces of literature, 19 case reports on pediatric Hoffa fractures were deemed relevant to this study (1–17). The research reports were critically evaluated for methodologies, findings, and contributions to identify research gaps and areas needing further investigation. A detailed table was compiled, including key literature details such as case authors, patient information, fracture types, injury mechanisms, surgical approaches, and outcomes.

According to the AO/OTA classification (13), unicondylar Hoffa fractures are classified as type 33-B3.2, while bicondylar Hoffa fractures are type 33-B3.3. Letenneur's study on cadaver specimens proposed a classification system for Hoffa fractures based on fracture location and direction of fracture lines, aiming to correlate fracture type with AVN of the femoral condyle (9). This study utilized the Salter-Harris classification to categorize eight cases of pediatric epiphyseal fractures, as demonstrated in the Letenneur score presented in Table 1. The literature review showed detailed Hoffa fractures in children, including fracture types, mechanisms of injury, surgical approaches, and outcomes. Treatment methods varied, with successful treatments of lateral and medial femoral condyle fractures through open reduction and internal fixation with screws. Most cases reported satisfactory outcomes, with patients achieving full range of motion (ROM) and minimal pain. Based on the reviewed literature (1–17), lateral femoral condyle fractures accounted for approximately 36.8% of cases, conjoint fractures for 31.6%, and medial femoral condyle fractures for 31.6%. Also, surgical approaches included open reduction through medial or lateral peripatellar approaches, arthroscopic evaluation followed by open arthrotomy, and posterior lateral approaches, each contributing diversely to outcomes. Most cases showed positive results in terms of ROM, pain management, and joint stability. The literature review highlighted the limited number of clinical and case study reports, particularly those utilizing techniques like the Letenneur score (5, 6, 15–17).

**Table 1:** Detailed information of all cases.

No	Years	Gender	Side	Mechanism of injury	Can X-R detect fractures	CT findings and Salter-Harris classification	Days of injury	Surgical Approach	Types of fixation	Letenneur score	Fracture Displacement (mm)
1	10	Boy	Left	Car accident	Yes	Medial femoral condyle fracture; type IV	5	Medial PPA	One 4.0 mm and one 4.5 mm partially threaded cannulated screws; A-P and L-M screw	Good	3.2
2	7.2	Boy	Left	Car accident	No	Medial femoral condyle fracture; Type Ⅲ	3	Medial PPA	Two 4.0 mm partially threaded cannulated countersunk screws; A-P screw	Good	2.5
3	14.7	Boy	Right	Car accident	Yes	lateral femoral condyle fracture; type Ⅲ	6	Lateral PPA	Three 4.5 mm partially threaded cannulated countersunk screws; A-P screw	Good	4.0
4	11.1	Boy	Left	Car accident	Yes	lateral femoral condyle fracture; type Ⅲ	5	Lateral PPA	Three 4.5 mm partially threaded cannulated countersunk screws; A-P screw	Good	3.1
5	9	Girl	Right	Car accident	No	Medial femoral condyle fracture; type Ⅲ	4	Medial PPA	Two 4.0 mm partially threaded cannulated countersunk screws; A-P screw	Good	2.2
6	12.2	Girl	Right	Car accident	Yes	lateral femoral condyle fracture; type Ⅲ	5	Lateral PPA	Three 4.5 mm partially threaded cannulated countersunk screws; A-P screw	Good	3.0
7	8.7	Boy	Right	Car accident	Yes	lateral femoral condyle fracture; type IV	2	Lateral PPA	Two 4.0 mm partially threaded cannulated countersunk screws; A-P screw	Good	2.7
8	7.9	Boy	Right	Fall from height	Yes	Bicondylar Hoffa's fracture; type Ⅲ	2	Medial PPA + DLA	Four 4.0 mm fully threaded cannulated countersunk screws; A-P and L-M screw; two 1.5 mm Kirschner Wires	Fair	6.0

Demonstrated Letenneur score of Good, denoting a favorable outcome. However, one case with bicondylar fracture experienced limitations in functional knee activity (100-0°), dysplasia of the distal femur, a 0.8 cm femur shortening, and a mechanical lateral distal femoral angle (mLDFA) discrepancy (92° compared to intact side's 88°). PPA, parapetallar approach; DLA, direct lateral approach. Table 1 indicates the analysis of detailed information on all cases.

There still exists a gap regarding a standardized treatment protocol for Hoffa fractures in the pediatric age group, notwithstanding earlier works done. The management of Hoffa fractures is largely inconsistent among studies as factors such as surgical methods, fixation, and rehabilitation practices vary. Gaps remain in the literature about long-term results and optimal management for pediatric Hoffa fractures. The current literature does not provide adequate information on how effective the various surgical treatments are or how they affect children's growth and functional joint activity. The anatomical specifics of the pediatric population, for example, an open growth plate, require that a special surgical approach be devised to allow for the anatomical reduction and preservation of growth possible (6). Hence, dealing with all the complexities of treating Hoffa fractures in children is highly necessary to prevent growth alterations and promote proper joint function.

The current study aims to improve the knowledge of pseudoparalytic Hoffa fractures, surgical management protocols, and clinical outcomes. This study aims to gather information from existing literature and case studies and provide an overview of differing surgical techniques and their successes to enable the clinician's evidence-based choices. These interesting findings widen the knowledge of pediatric surgery by using the Letenneur score technique to fill this gap and improve physicians' understanding of effective treatment protocols for Hoffa fractures in children. Finally, various surgical treatments in light of literature and case studies will provide an important base for clinicians to manage these injuries in pediatric patients better, which should help promote improved patient outcomes (5, 6, 15–17).

## Materials and methods

The study adhered to ethical principles and received approval (no. KY 2023-014-01) from the Sichuan Provincial Orthopedic Hospital Pediatrics Department. Informed consent for participation was obtained from the parents or legal guardians of all participants under 16. Informed consent was obtained from all participants or their legal guardians before inclusion. Conducted between January 2003 and January 2021, the study ensured adequate follow-up to assess long-term outcomes and complications. During the study, preoperative CT examinations classified fractures according to Salter-Harris type III or IV (9–12). Surgical intervention was indicated based on literature suggesting that these fractures typically involve the growth plate (9–12). The surgical approach was tailored to the fracture characteristics, ensuring optimal exposure and fixation based on the fracture type and displacement (1–19). Common surgical approaches included open reduction and internal fixation (ORIF) using screws, plates, or other fixation devices to stabilize the fractures and promote healing (8–10).

### Surgical approach selection

In unicondylar fractures, the PPA offers direct access to the femoral condyle while limiting soft tissue damage. The PPA was appropriate for lateral and medial Hoffa fractures as they allow quick reduction and fixation. In contrast, bicondylar fractures required better exposure by combining the DLA with the medial PPA. This was deemed the best surgical choice regarding the optimal view and stabilization that could be achieved because of the heterogeneous presentations associated with bicondylar fractures. This choice warranted a tailored approach towards achieving adequate stability in fixation while preserving knee joint function.

All procedures were performed under general anesthesia, with proper regional anesthesia during the surgical phase. Patients were placed supine with the affected limb flexed to 30°. Standard disinfection and draping procedures were followed, with a sterile tourniquet applied proximally. For unicondylar fractures, an incision was made either above or below the joint plane to facilitate patellar retraction and full exposure of the fracture site for accurate anatomical reduction. A direct lateral approach (DLA) and medial PPA were used in the only case of bicondylar fracture. Care was taken to preserve soft tissue on joint entry to minimize vascular compromise to the femoral condyle.

### Fixation method and screw trajectory

The stabilization policy was based on stabilizing and orienting the fracture. Initially, direct vision was used to reduce the fracture and maintain alignment by securing it with 1.5 mm Kirschner wires perpendicular to the fracture plane. The alignment of the bone was confirmed fluoroscopically before introducing 2–4 cannulated compression screws (4.0 or 4.5 mm) following either anterior-to-posterior (A-P) or posterior-to-anterior (P-A) trajectories according to the fracture configuration. A-P screw placement was preferred in cases of minimal displacement of the fracture for optimal fixation without impinging upon the articular surface. On the other hand, P-A screws were selectively used in cases of fractures that needed more stability, particularly those with significant comminution or bicondylar involvement. This enabled adequate fracture site compression, promoting healing while reducing hardware prominence in the joint space.

**Figure 1:**
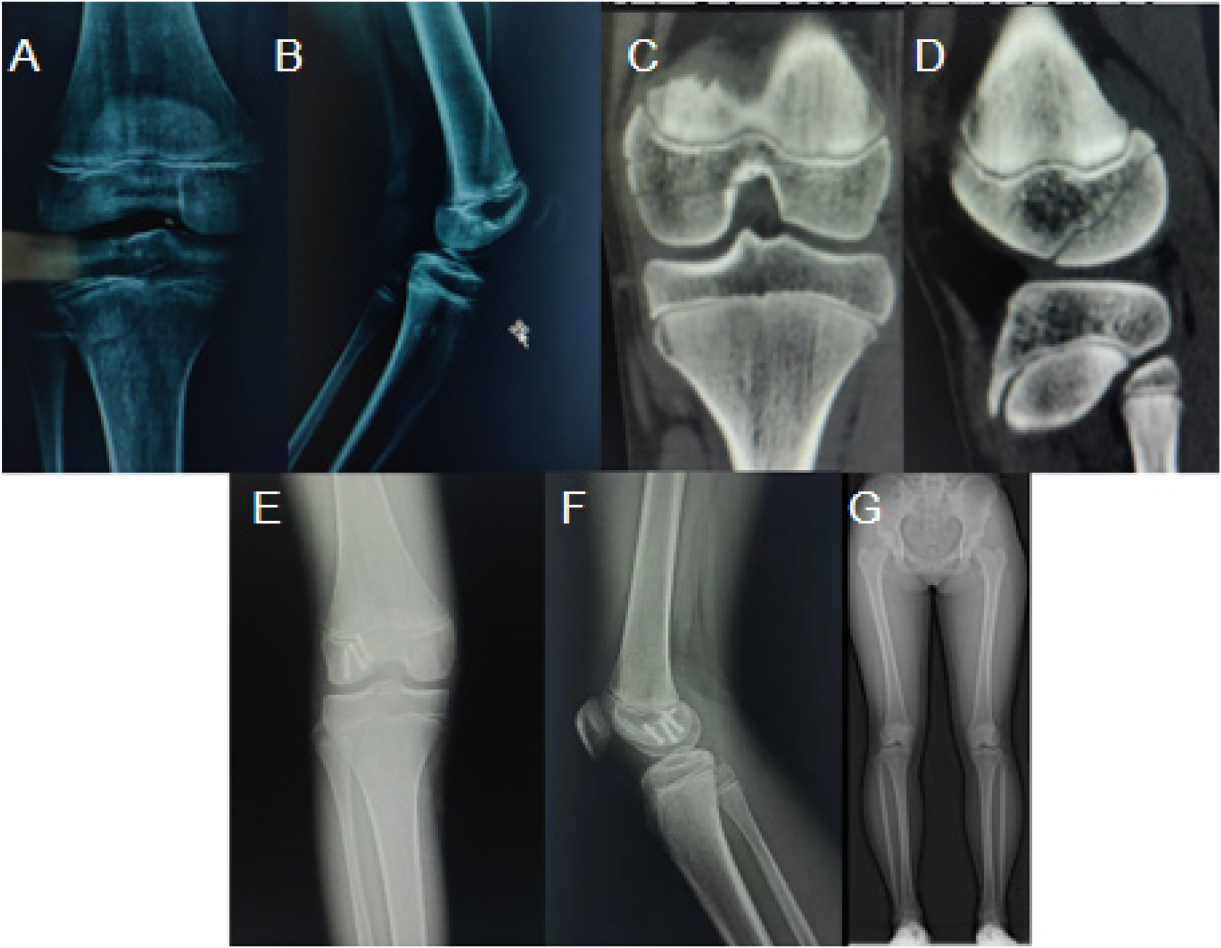
A case of a 12-year-old girl with a right lateral femoral condyle fracture from a car accident. **(A, B)** Preoperative X-rays showing the break; **(C, D)** preoperative CT images for detailed conditions; **(E, F)** Postoperative X-rays showing bone healing; **(G)** Full-length X-ray of both lower limbs three years post-surgery.

**Figure 2:**
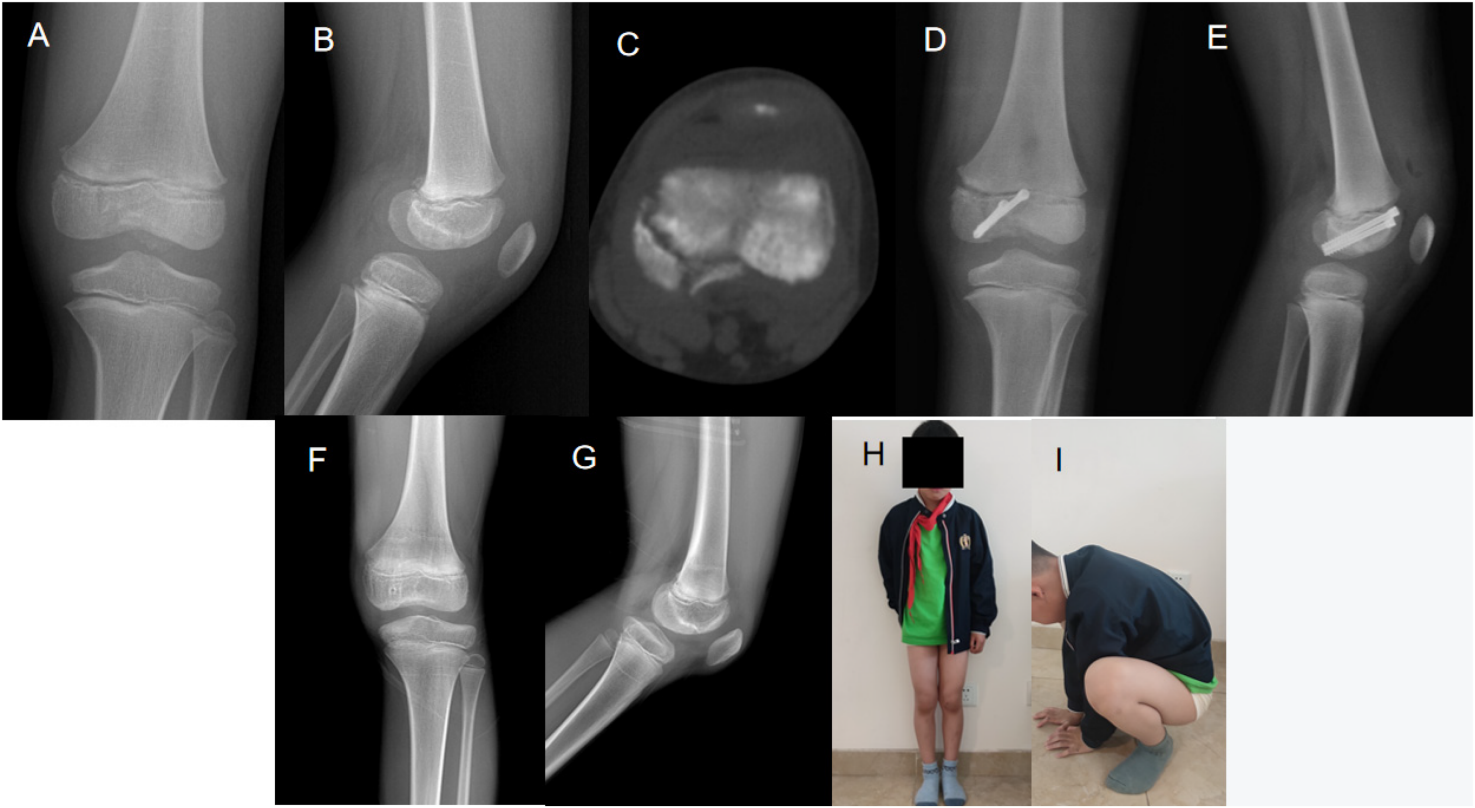
A 7.2-year-old boy with left medial femoral condyle fracture from a vehicle accident: **(A)** X-rays preoperative showing fracture; **(B)** CT exam preoperative for the evaluation of injury; **(C)** Postoperative images showing two 4.0 mm screws for fixation; **(D)** Two years post-operative X-rays showing healing process; **(E)** Postural images 18 months post-surgery showing functional recovery; **(F, G)** Post-operative X-rays showing alignment; **(H, I)** Clinical images showing functional results and mobility.

**Figure 3:**
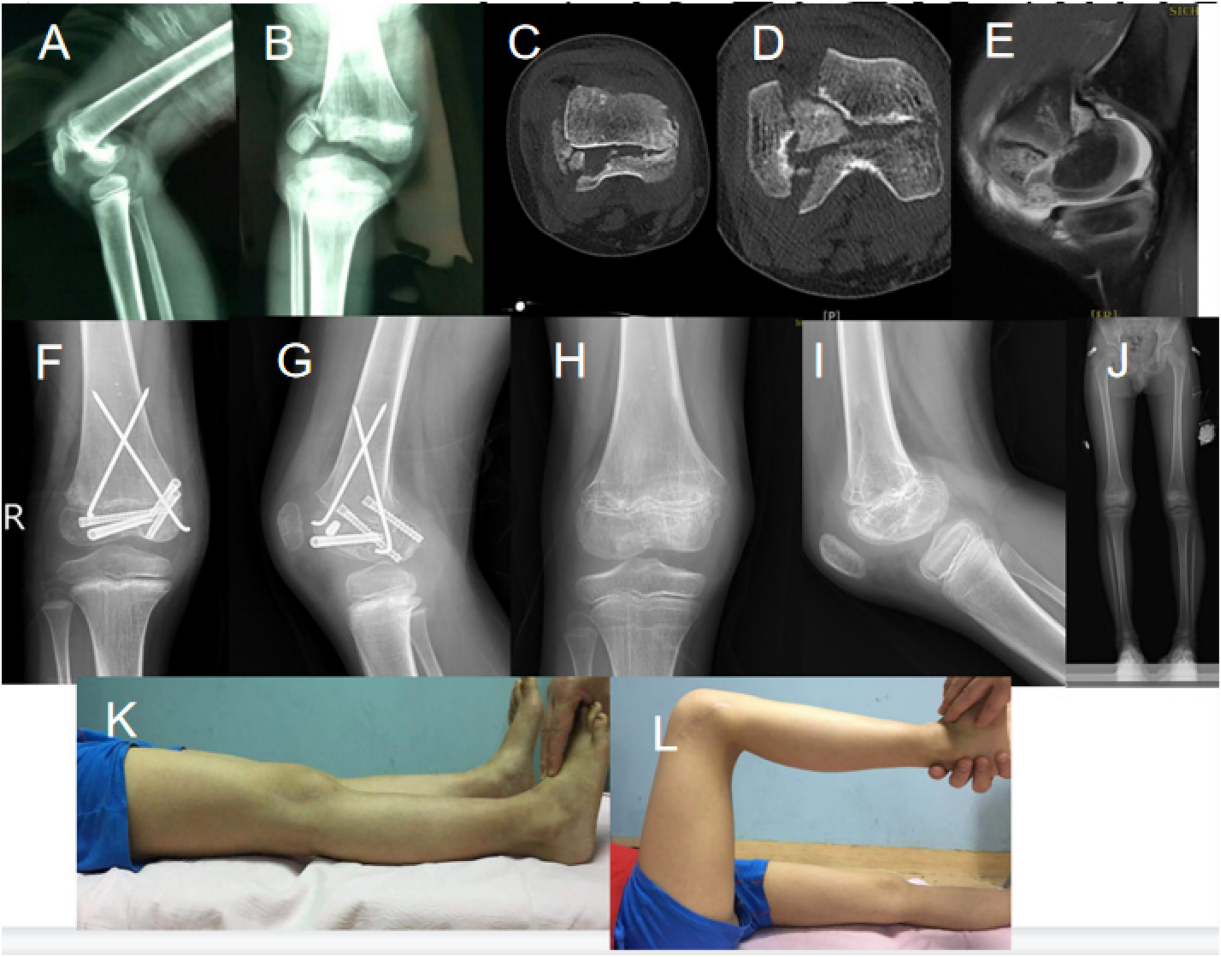
An injured 7.9-year-old boy with bicondylar Hoffa's fracture arising from a high fall injury. **(A, B)** X-rays of the fracture taken in a preoperative state; **(C, D, E)** CT and MRI performed in a preoperative state and establishing the injury severity; **(F, G)** Post-operative X-rays showing internal fixation; **(H, I)** X-rays demonstrating bone healing during follow-up; **(J)** full-length X-ray of both lower limbs; **(K, L)** clinical images showing functional recovery and range of motion.

The wound closer followed the Metz White surgical protocol described in Figures 1–3.

Finally, postoperative results were quantified using a Letenneur score (9, 17), classifying outcomes into Good, Fair, and Bad based on knee range of motion, stability, pain levels, and need for auxiliary walking (Table 1). This systematic approach enables structured assessments of functional rehabilitation, pain management, joint stability, and postoperative development. The study ensured a comprehensive evaluation of surgical outcomes and patient progress, enhancing the replicability and comparability of the study outcomes (9, 17).

## Results

This study is centered on the treatment outcomes in children with Hoffa fractures and the various complication analyses of these fractures. On average, bone union took 15.8 weeks post-surgical fixation, with none suffering from nonunion or significant complications.

The Letenneur score, which assigns scores concerning knee function and overall appearance, indicated that out of the confirmed cases, the fractures among seven patients were managed according to the good rating (Table 1). This is testimony to the effectiveness of treatment, whereby anatomical alignment and restoration of functional mobility were achieved without complications, such as premature closure of the upper tibia epiphysis.

Case analysis showed that a full recovery had been achieved in the group with fractures where the displacement was ≤2 mm (*n* = 6). The case with displacement >2 mm showed a less favorable functional outcome. The bicondylar fracture displayed restricted knee function, with a maximum active range of motion of 100°. In addition, the patient displayed distal femur dysplasia with shortening of the femur by 0.8 cm. The angle between the mechanical lateral distal femur (MLDF) was not equal (92° vs. 88° on the intact side). Despite all these handicaps, this case was scored as Fair by the Letenneur scoring system.

### Case descriptions

#### Case 1: right lateral femoral condyle fracture

This case was of a 12-year-old girl who sustained a right lateral femoral condyle fracture due to a car accident. The severity of the fracture was comprehensively demonstrated preoperatively on X-rays (Figure 1A and 1B) and CT imaging (Figure 1C and 1D). Postoperative X-rays (Figure 1E and 1F) and full-length lower limb radiographs taken three years after surgery (Figure 1G) confirmed successful fracture healing and satisfactory long-term functional recovery.

Notably, functional images were unavailable for this case due to the loss of follow-up after the last radiographic assessment. Further, despite several attempts, little postoperative clinical evaluation or functional imaging could be obtained.

#### Case 2: left medial femoral condyle fracture

This 7.2-year-old boy sustained a left medial femoral condyle fracture consequent to a vehicular accident. The preoperative X-rays (Figure 2A and 2B) and CT images (Figure 2C) confirm the injury. Post-operative imaging (Figure 2D, 2E) showed successful placement of surgical screws and progressive healing. The follow-up X-rays at two years post-surgery (Figure 2F and 2G) and clinical posture assessment at 18 months (Figure 2H and Figure 2I) demonstrated continued recovery and improved function.

#### Case 3: bicondylar Hoffa fracture

A 7.9-year-old boy was treated for a bicondylar Hoffa fracture as a result of a fall from a height. Imaging prior to surgery, including X-rays (Figure 3A and Figure 3B), CT (Figure 3C and Figure 3D), and MRI (Figure 3E), confirmed the extent of the fracture and involvement of adjacent anatomical structures. Postoperative X-rays (Figure 3F and Figure 3G) showed that internal fixation was done using screws and Kirschner wires. Follow-up X-rays done at 26 months (Figure 3H and Figure 3I) showed persistent dysplasia of the femur with shortening and varus deformity of the knee joint. Despite surgery, there were still limits in the range of motion and corresponding difficulties restoring normal knee function revealed from a functional analysis performed in clinical evaluation (Figure 3K and Figure 3L).

## Discussion

Hoffa's fractures are traumatic injuries primarily affecting the distal femur. They typically result from high-energy incidents and are more commonly observed among young adults (20, 21). This study investigates the clinical implications and management of Hoffa's fractures in children, highlighting their distinct characteristics compared to adult cases. Unlike adults, children with Hoffa fractures generally do not present with concurrent cruciate ligament or meniscus injuries, which is attributed to the greater strength of ligaments relative to their epiphyses (6, 19, 22).

The mechanisms leading to Hoffa's fractures vary, with some studies emphasizing direct impact on a flexed knee, while others suggest a combination of shear and torsional forces (23). Lewis et al. propose that these fractures occur due to axial stress on the femoral condyle during knee flexion beyond 90°, possibly exacerbated by slight valgus positioning (14). The direction of varus or valgus pressure significantly influences the fracture pattern, with single lateral Hoffa fractures being more prevalent due to the intrinsic valgus angle of the human knee joint (14).

Hoffa fractures should be differentiated from other pediatric femoral injury types, especially Salter-Harris IV and V distal femoral physeal fractures. Whereas Hoffa fractures are considered intra-articular, coronal-plane fractures of the femoral condyle, Salter-Harris IV and V fractures involve the growth plate, making them more physical injuries. The two fracture types have different mechanisms of injury; Salter-Harris IV and V fractures usually occur when axial loading forces are transmitted through the epiphysis and metaphysis, causing physical disruption. Hoffa fractures are mostly caused by high-energy forces with shearing and compressive components acting upon a flexed knee; they might affect the articular surface rather than the physis. Apart from mechanisms of trauma, treatment strategies also differ in the sense that Salter-Harris fractures require physical preservation techniques, such as minimally invasive leverage reduction, as espoused by recent literature (15). Therefore, knowledge of the differences above is key to determining the appropriate management method and predicting the long-term outcome.

We studied only children under 14 with different patterns of fractures. Although lateral condylar fractures were the most common, contrary to reports from previous studies (14, 23), medial condylar fractures were frequently documented, indicating some variations in the knee joint position at the time of injury (5, 6). The diagnostic functions of CT scanning were extremely valuable in identifying Hoffa fractures when the displacement was either negligible or non-existent, and this allegedly could not be assessed adequately by conventional radiography. MRI was also useful in assessing associated soft tissue injuries; however, within the spectrum of injuries described above, MRI is often avoided unless there is suspicion of neurovascular, ligamentous, or meniscal damage (24–27).

Surgical intervention is usually indicated in Hoffa's fractures in children because of the high risk of late displacement and poor function of the joint following non-surgical management (4). Anatomical reduction, stable internal fixation, and early mobilization are crucial in optimizing long-term outcomes with reduced risk of complications such as avascular necrosis (AVN) of the femoral condyle (28). The anterior-to-posterior screw fixation is now emerging as a preferred method in pediatric cases, showing good results for the reduction and stability of the fracture (19, 29, 30). In complex cases such as bicondylar fractures, combining PPA with other fixation methods, such as the DLA, may be necessary to attain the best functional outcomes, as seen in our case study.

While we used the Letenneur score to assess outcomes in our study, we do not intend to prove its reliability. It is also important to mention that this study has a small sample size and does not include a control group, which limits our ability to draw conclusions regarding the applicability of this system as a universal evaluation for pediatric Hoffa fractures.

Arthroscopic techniques have gained more recognition as an effective approach for intra-articular fractures, including Hoffa fractures. In this regard, our study did not employ arthroscopy due to the complex nature of Hoffa fracture and the desire to use open reduction techniques with stable fixation. Subsequent research could investigate the feasibility and advantages of arthroscopic-assisted fixation in pediatric Hoffa fractures, especially in the case of minimally displaced ones.

While this study may give a new orientation toward surgical approaches and their outcomes, future researchers and practitioners have yet to sort out some limitations. The long-term heterogeneity of cases raised challenges in maintaining uniformity in surgical techniques and assessments. The low number of complex bicondylar and comminuted fractures limits the generalizability of our results. The absence of a control group of non-operatively treated patients diminishes the conclusiveness regarding the superiority or otherwise of surgical against conservative management for such conditions.

Currently, there is no standardized management protocol for pediatric Hoffa fractures. Further investigations should focus on accumulating evidence leading to the development of optimal treatment algorithms, using both surgical and conservative measures where applicable. Multicenter studies with more patients over long-term functional outcomes analysis are necessary to formulate standardized management protocols.

Although the number of cases in this study is small, it provides new insight into the surgical management of pediatric Hoffa fractures. It further contrasts itself with previous studies in giving structured comparisons of the various approaches and their outcomes, seeking to address the particular courses of action intended for the different presentations of Hoffa fractures.

Thus, surgical treatment for Hoffa fractures in children must be individualized, with rigorous support from advanced imaging studies in making exact diagnoses and performing surgeries that preserve joint function and minimize long-term sequelae. Future investigations will improve treatment algorithms and outcomes in these challenging orthopedic problems.

## Conclusion

In conclusion, this study, an 8-case series that aims to explore the surgical management of Hoffa fractures in pediatric patients, provides useful insights into the surgical techniques for managing these fractures. Unfortunately, since no cases of conservative treatment were included, a proper comparative study of surgical and conservative management was not possible. According to the CT scan, imaging is the preferred modality for assessing fracture morphology and making treatment decisions. The findings of this study would warrant further modification and an understanding of the Letenneur scoring technique for customizing treatment plans for children with Hoffa fractures.

This study eludes the standardization of set management systems, as there is room for differences in fracture patterns and management modes. Future work must involve multi-institutional studies with larger patient numbers to develop consensus guidelines on pediatric Hoffa fractures incorporating surgical and conservative management paths, wherever applicable. This study indicates that while most intervention techniques may be minimally or not invasive, they are compelling in their medical significance; however, further investigation is necessary to standardize treatment protocols and improve patient outcomes. As we advance our innovative techniques, further studies and combined efforts are necessary to improve Hoffa fracture treatment in children to assist in clinical decision-making and reproducibility.

## Data Availability

The raw data supporting the conclusions of this article will be made available by the authors, without undue reservation.
